# Understanding type 2 diabetes mellitus screening practices among primary care physicians: a qualitative chart-stimulated recall study

**DOI:** 10.1186/s12875-017-0623-3

**Published:** 2017-04-04

**Authors:** Dina Hafez, Daniel B. Nelson, Evan G. Martin, Alicia J. Cohen, Rebecca Northway, Jeffrey T. Kullgren

**Affiliations:** 1 VA Ann Arbor Healthcare System, 2800 Plymouth Road, Building 14, Room G100-36, Ann Arbor, MI 48109-2800 USA; 2 Robert Wood Johnson Foundation Clinical Scholars Program, University of Michigan, Ann Arbor, MI USA; 3 University of Michigan Medical School, Ann Arbor, MI USA; 4 University of Michigan Institute for Healthcare Policy and Innovation, Ann Arbor, MI USA

**Keywords:** Type 2 diabetes mellitus, Prediabetes, Diabetes prevention, Primary care, Preventive care, Communication, lifestyle counseling

## Abstract

**Background:**

Early diagnosis and treatment of prediabetes and type 2 diabetes mellitus (T2DM) can prevent future health problems, yet many individuals with these conditions are undiagnosed. This could be due, in part, to primary care physicians’ (PCP) screening practices, about which little is known. The objectives of this study were to identify factors that influence PCPs’ decisions to screen patients for T2DM and to characterize their interpretation and communication of screening test results to patients.

**Methods:**

We conducted semi-structured chart-stimulated recall interviews with 20 University of Michigan Health System (UMHS) primary care physicians. PCPs were asked about their recent decisions to screen or not screen 134 purposively sampled non-diabetic patients who met American Diabetes Association criteria for screening for T2DM. Interviews were audio-recorded, transcribed, and analyzed using qualitative directed content analysis. Data on patient demographic characteristics and comorbidities were abstracted from the electronic health record.

**Results:**

The most common reasons PCPs gave for not screening 63 patients for T2DM were knowledge of a previously normal screening test (49%) and a visit for reasons other than a health maintenance examination (48%). The most common reasons PCPs gave for screening 71 patients for T2DM were knowledge of a previously abnormal screening test (49%), and patients’ weight (42%) and age (38%). PCPs correctly interpreted 89% of screening test results and communicated 95% of test results to patients. Among 24 patients found to have prediabetes, PCPs usually (58%) recommended weight loss and increased physical activity but never recommended participation in a Diabetes Prevention Program or use of metformin.

**Conclusions:**

Previous screening test results, visit types, and patients’ weight and age influenced PCPs’ decisions to screen for T2DM. When patients were screened, test results were generally correctly interpreted and consistently communicated. Recommendations to patients with prediabetes could better reflect evidence-based strategies to prevent T2DM.

**Electronic supplementary material:**

The online version of this article (doi:10.1186/s12875-017-0623-3) contains supplementary material, which is available to authorized users.

## Background

Type 2 diabetes mellitus (T2DM) affects 25.1 million US adults and is a major cause of cardiovascular disease, kidney disease, blindness, and lower extremity amputation [1]. An additional 86 million individuals – one third of the US adult population – are estimated to have prediabetes [1], which if left untreated will result in 5–10% of these individuals progressing to T2DM each year [2].

Fortunately, T2DM and its complications can often be prevented through early detection and treatment of T2DM [3] and prediabetes [4, 5]. Several national initiatives are now aiming to identify more individuals with these conditions and connect them to evidence-based treatments. For example, in 2015 the US Centers for Disease Control and Prevention (CDC) and the American Medical Association (AMA) launched “Prevent Diabetes STAT: Screen/ Test / Act Today,” a joint initiative that encourages diabetes prevention through screening for T2DM and referral to a Diabetes Prevention Program (DPP) [6]. Also in 2015, the United States Preventive Services Task Force (USPSTF) updated its recommendation for screening of adults for T2DM to include behavioral counseling interventions for those with abnormal results [7]. Most recently, the Centers for Medicare and Medicaid Services announced Medicare would cover the DPP for beneficiaries with prediabetes [8], joining the growing numbers of private insurers already doing so [9].

The promise of these national initiatives to improve population health hinges on the early identification and management of T2DM and prediabetes, which often begins in primary care practices through tests to screen for T2DM and communication of these test results to patients. Therefore, decisions primary care physicians make about whom to screen for T2DM, interpretation of screening test results, and communication of these results to patients can have important implications. For example, if clinicians do not accurately perceive risk factors for T2DM or if they incorrectly interpret screening test results, they may fail to identify at-risk patients. Further, clinicians may not communicate T2DM screening test results to patients or they may not provide evidence-based treatment recommendations, which can affect patients’ perceptions of risk and their decisions about preventive behaviors [10]. If these deficiencies are widespread, they could help explain why more than 80 million individuals with T2DM and prediabetes are unaware they have these conditions [11].

Despite the population health implications of primary care physicians’ T2DM screening practices, little is known about how primary care physicians decide which patients to screen for T2DM, what screening tests they choose, how they interpret screening test results, and what they communicate to patients about these results. The objective of our study was to identify factors that influence primary care physicians’ decisions to screen patients for T2DM and to characterize primary care physicians’ interpretation and communication of screening test results to patients.

## Methods

### Study design

Our team, which consisted of 3 primary care physician health services researchers, 1 fulltime primary care clinician, and 2 preclinical medical students, invited Internal Medicine physicians (*n* = 50), Family Medicine physicians (*n* = 39), nurse practitioners (*n* = 2), and physician assistants (*n* = 5) from 14 UMHS primary care practices to participate in a study about T2DM screening practices. We excluded trainees and clinicians with less than 0.5 full time equivalents devoted to outpatient clinical practice. Study invitation letters were sent by email, and informed providers that the primary aim of this study was to explore the factors that influence providers’ decisions to screen for T2DM.

Among clinicians who consented to study participation we conducted chart-stimulated recall (CSR) interviews, a methodology used to assess clinical decision-making processes [12, 13]. During CSR interviews, a clinician uses his or her own documentation to answer questions and explain the rationale for specific clinical decisions. A significant strength of this approach – which has been used to examine physician decisions about screening for colorectal cancer [14] and screening for prostate cancer [15] – is that it examines a physician’s own recent clinical decisions, which could provide more valid data on their clinical decision making than assessments of how they might respond to hypothetical clinical scenarios.

### Sample

Twenty-five physicians responded to our recruitment e-mail and agreed to participate in our study. We planned to conduct a minimum of 20 interviews with additional interviews to be conducted only if data saturation was not achieved at this point. Prior to each CSR interview, one investigator (DH) reviewed in the electronic health record (EHR) each physician’s clinical encounters from the previous 2 weeks to identify visits for non-diabetic patients 45 years of age and older who were candidates for screening for T2DM at their visit and thus eligible for inclusion in the CSR interviews. We focused on patients 45 years of age and older for 2 reasons. First, the American Diabetes Association (ADA) recommends screening all individuals over the age of 45 for T2DM regardless of other risk factors; therefore an age-based focus allowed us to easily identify patients for whom the ADA recommends periodic T2DM screening. Second, this age-based criterion was more feasible to implement in the context of the EHR than other more detailed criteria for which the ADA recommends screening for T2DM, such as being less than 45 years of age and overweight with at least one additional risk factor for T2DM [16].

While both the ADA and the USPSTF recommend routine screening for T2DM among at-risk individuals, we used the ADA’s screening criteria because at the time of our interviews they were broader than the 2008 USPSTF criteria [17]. The USPSTF has since issued revised screening guidelines (disseminated in October 2015), but these were released after completion of all CSR interviews. We did not sample patients who had already been screened for T2DM within the previous year, as most non-diabetic patients do not need to be screened more than annually [16] and this study was not designed to explore reasons for over-screening.

To facilitate discussion of the range of factors that influence physicians’ decisions to screen for T2DM, we selected in approximately equal numbers encounters in which patients appeared (using the available information in the EHR) to have been screened for T2DM or not screened for T2DM. We classified the following as screening tests for T2DM, based on the ADA guidelines: hemoglobin A1c (HbA1c) tests, oral glucose tolerance tests (OGTTs), and fasting blood glucose (FBG) tests [16]. Because blood glucose tests at our institution are not routinely labeled as “fasting,” we classified blood glucose tests (drawn either in isolation or as part of a metabolic panel) as being fasting if they were obtained before 11 a.m. or drawn at the same time as a lipid panel that had been labeled in the EHR as fasting.

We also sampled in approximately equal numbers encounters in which patients were seen for either health maintenance examinations (HMEs) or return visits (RVs). We focused on both HMEs and RVs because there is debate about the utility of annual HMEs [18–20] and physicians have been encouraged to consider providing opportunistic preventive screenings at non-HME visits [21–23]. We excluded urgent care visits because in our institution these are typically focused, single-problem visits in which patients are commonly seen by clinicians other than their own primary care clinician.

Using this approach, for each clinician we aimed to select 12 encounters (i.e., roughly 3 each for HMEs with screening, HMEs without screening, RVs with screening, and RVs without screening) from the previous 2 weeks to potentially be discussed during the CSR interview. A goal of 12 encounters for each physician provided the opportunity to skip a particular encounter during the interview in the event that the clinician did not recall the encounter, while still permitting discussion of at least 6 separate patient encounters which has previously been shown sufficient to yield a valid and reliable assessment of practice patterns [24].

Interviews were conducted by trained preclinical medical students (DN, EM) and lasted approximately 30 min in duration. During the CSR interviews, physicians were first asked whether they screened each patient for T2DM and what factors influenced this decision. Our questions about reasons for the physician’s decision were guided by whether the physician stated they had or had not screened the patient for T2DM, rather than how the patient had been classified in our purposive sampling. For example, if it appeared from the EHR that the physician had screened the patient for T2DM, but the physician stated that they had not screened the patient for T2DM, then the physician was asked why they had not screened the patient for T2DM. Conversely, if a patient had been sampled as having not been screened (e.g., because they had a random glucose), but the physician said they had screened the patient for T2DM, we asked the physician why they screened the patient (even though the test chosen was not an ADA-recommended test). This approach allowed us to focus on physicians’ decision making processes irrespective of the outcome of that process.

When a screening test result was available for review (i.e., there was a lab result for a screening test that had been ordered at that visit), physicians were asked how they interpreted the result, whether and how they communicated the result to the patient, and what they communicated to the patient about the result. Following the CSR interview, physicians were also asked general questions regarding barriers to T2DM screening. Physicians had access to their EHR documentation during the interview. The interview guide was derived from previous CSR studies [14, 15] and is provided in the Additional file 1.

For each interview, physicians received a $50 gift card. The study was approved by the Institutional Review Board at the University of Michigan Medical School.

### Analysis

All interviews were audio-recorded and transcribed verbatim. Three members of the research team (DH, DN, JK) independently reviewed a subset of transcripts. The transcripts were de-identified prior to data analysis to minimize the potential for biased interpretation of the data. Codes and definitions were generated during consensus conferences using directed content analysis [25]. Specifically, initial codes were created to reflect the main topics in the interview guide (e.g. decision to screen or not screen patients for T2DM), and additional codes were subsequently generated to reflect the patterns and themes that emerged from the data [26]. Once the coding scheme was established, two investigators (DH, DN) independently coded each transcript. These investigators then met to review their coding and resolve all differences. Few new themes emerged after coding 12 transcripts and no new themes emerged after coding 16 transcripts. Given that we reached data saturation [27, 28], we did not conduct additional interviews following the 20 interviews. All transcripts were coded in Dedoose, a software program for qualitative analysis.

The following data were abstracted from the EHR for each patient after the interviews were completed: age; sex; race; ethnicity; BMI; risk factors for T2DM documented in the patient’s problem list such as hypertension, hyperlipidemia, cardiovascular disease, prediabetes, history of gestational diabetes or polycystic ovarian disease.

## Results

Twenty primary care physicians (14 Internal Medicine and 6 Family Medicine) across 10 clinic sites participated in the study. There were no nurse practitioners or physician assistants who responded to our invitation.

These 20 physicians discussed a total of 156 patients who met our study inclusion criteria (mean 7.8 patients per physician) during the CSR interviews we conducted between January 2015 and July 2015. We then limited our analyses to the 63 patients who physicians stated they did not screen for T2DM and the 71 patients who physicians stated they screened for T2DM. We excluded 22 patients from our analyses because at the sampling stage they appeared to have been screened for T2DM with a blood glucose drawn prior to 11 a.m., but during the interview physicians indicated they did not screen the patient for T2DM and rather had ordered the blood glucose as part of an electrolyte panel to monitor kidney function.

A total of 134 patients were included in our analysis. In general, the patients who were screened for T2DM were had a higher BMI and a greater prevalence of prediabetes compared to patients who were not screened. Additionally, screening was more likely to occur during HMEs than RVs. Patient characteristics, stratified according to whether they had been screened for T2DM during the discussed encounter, are summarized in Table 1.

**Table 1:** Patient characteristics (*n* = 134)

Characteristics	Screened for T2DM (*n* = 71)	Not screened for T2DM (*n* = 63)	*p*-value^a^
	Mean (SD) or n (%)		
Age (years)	57.5 (9.6)	61.4 (12.2)	0.041
BMI (kg/m^2^)	31.4 (6.6)	28.6 (5.7)	0.012
Female	34 (47.9)	39 (61.9)	0.10
Race^b^			
White	57 (80.3)	57 (90.5)	0.23
Black	7 (9.9)	2 (3.2)	
Asian	3 (4.2)	1 (1.6)	
Hispanic ethnicity^c^	3 (4.23)	1 (1.6)	0.37
Visit type^d^			
Health maintenance examination	48 (67.6)	19 (30.2)	<0.001
Return visit	23 (32.4)	44 (69.8)	
Comorbidities^e^			
Hyperlipidemia	38 (53.2)	25 (39.7)	0.11
Hypertension	28 (39.4)	21 (33.3)	0.46
Cardiovascular disease	3 (4.2)	3 (4.8)	0.88
Prediabetes^f^	15 (21.1)	2 (3.2)	0.002

^a^ *p*-values were derived using logistic regression for continuous variables and Chi-squared test ﻿for categorical variables

^b^Race was listed in the EHR as “other” or “not reported” for 6 patients (4.5%)

^c^Ethnicity was listed in the EHR as “unknown” or “not reported” for 12 patients (9.0%)

^d^“Return visits” refer to problem-focused visits that range from 15 to 30 min in duration. “Health maintenance examinations” refer to prevention-focused visits that range from 40 to 45 min in duration

^e^The list of co-morbidities was selected based on the American Diabetes Association’s criteria for screening for type 2 diabetes mellitus. There were no patients in our sample who had a documented history of 2 other ADA criteria for screening: polycystic ovarian syndrome and gestational diabetes mellitus

^f^Patients were classified as having prediabetes if they had one or more of the following diagnoses listed on their EHR problem list: prediabetes, impaired glucose tolerance, impaired fasting glucose, or borderline diabetes

### Screening decisions

For the 63 patients who were not screened for T2DM, the most common reasons for not screening that physicians cited were knowledge of a previous normal screening test (49%) and the visit being for a reason other than a health maintenance examination (48%). Clinicians frequently cited more than one reason for not screening, and additional reasons are shown in Table 2.

**Table 2:** Physician-identified reasons for not screening for type 2 diabetes mellitus (*n* = 63)

Reasons	n (%)^a^
Previously normal T2DM screening test result(s)	31 (49.2)
Return visit	30 (47.6)
Normal weight	6 (9.5)
Future health maintenance examination	4 (6.4)
Normal blood pressure	2 (3.2)
Did not believe screening indicated based on guidelines	2 (3.2)
Younger age	2 (3.2)
Other^b^	12 (19.1)

^a^Number of times code occurred and frequency of occurrence among the 63 patients who were not screened. Codes were not mutually exclusive and therefore the percentages sum to more than 100

^b^Includes physician not being the patient’s primary care physician; patient reported recent normal labs obtained elsewhere; patient preference to avoid a blood draw; patient enrolled in hospice

For the 71 patients who were screened for T2DM, the most common reasons for screening that physicians cited were knowledge of a previously abnormal screening test for T2DM (49%), the patient being overweight or obese (42%), and patient age (38%). Two physicians specifically mentioned age ≥ 45 years as a screening criterion; others referenced the patient’s actual age (e.g. “she is 60 years old”) or noted that “the risk of diabetes goes up as we get older.” Clinicians often cited multiple reasons for screening, and these additional reasons are shown in Table 3.

**Table 3:** Physician-identified reasons to screen for type 2 diabetes mellitus (*n* = 71)

Reasons	n (%)^a^
Previously abnormal screening test result(s)	35 (49.3)
Overweight or obesity	30 (42.3)
“Older age”	27 (38.0)
Hypertension	18 (25.4)
Hyperlipidemia	11 (15.5)
Health maintenance examination	8 (11.3)
Family history of T2DM	6 (8.5)
Sedentary lifestyle	4 (5.6)
History of cardiovascular disease	3 (4.2)
Race/ethnicity	3 (4.2)
Tobacco use	3 (4.2)

^a^Number of interviews in which the theme emerged and the frequency of occurrence using total number patients who physicians intended to screen (71) in the denominator

Among the 71 patients who were screened for T2DM, the most commonly ordered screening tests were a laboratory HbA1c (*n* = 29, 40.8%) and a fasting blood glucose (*n* = 23, 32.4%). Four patients (5%) were screened with a point-of-care HbA1c, and 8 patients were screened with a random blood glucose (i.e., a blood glucose drawn after 11 a.m.). Seven patients (9.9%) had more than one screening test ordered (i.e., both a fasting blood glucose and an HbA1c). No physician ordered an OGTT to screen for T2DM. Physicians reported ordering an HbA1c test for reasons such as: “it is more familiar and comfortable,” “it is a little more reliable,” “patients don’t need to be fasting” and “it gives a better [overall] picture.” Physicians ordered a fasting blood glucose – either in isolation or as part of a metabolic panel – if the patient had already been fasting for another test (e.g., a fasting lipid panel) or if the physician wanted to concurrently evaluate other components of the metabolic panel (e.g., electrolytes or renal function).

### Interpretation of screening test results

Of the 71 patients for whom screening tests for T2DM were ordered, 50 had screening test results available in the EHR for review at the time of the interview. Based on the ADA guidelines,[29] 24 patients had screening test results that showed prediabetes, and 26 patients had screening test results that were normal. None of the sampled patients were found to have a new diagnosis of T2DM. Physicians discussed 47 of the 50 available test results, and of these correctly classified the majority (42, 89%) of the screening test results.

### Communication of screening test results

Physicians communicated 95% of screening test results to patients. Most communications occurred by mailed letter or secure electronic messaging through the health system’s patient portal (69%). Communication occurred by phone when either the screening test or another laboratory study was abnormal (16%); such telephone communications were always performed by a nurse. Communication occurred during an office visit only if the patient had laboratory tests performed prior to the encounter (15%).

Among patients found to have prediabetes, the content of communications focused mostly on diet and exercise, often with offers of referral to a nutritionist. For example, one physician stated, “I told her…diet and exercise would be the key. [We] can also offer referral to our nutritionist for pre-diabetes counseling, if she would like.” In many cases, physicians’ recommendations to patients were general and lacked specific goals or targets. An example of this was a physician who recommended, “just to continue with, what we call…lifestyle modifications. You know, a little bit of exercise, healthy diet, stuff like that.” Some physicians minimized the significance of screening test results if they were at the lower end of the prediabetic range. For example, one physician said, “[The blood glucose] is slightly high at 101. What I told [him] is that he is just out normal range, but not in a concerning…urgent way, and that we’ll talk about it in his next visit.” Another said, “[The HbA1c] is stable from a year ago at 6.2%, which I feel is consistent with pre-diabetes and puts her at a high risk of developing diabetes…[I told her] that the lab looks stable…I kept it very short and sweet. I probably could have said something more about lifestyle.”

Communications to patients with prediabetes never included specific, evidence-based treatment recommendations such as DPP participation or pharmacotherapy with metformin [30]. One physician said that “we talked about the diabetes prevention trial, and we set 150 min of exercise for her goal [per] week,” but she did not specifically recommend or refer the patient to a DPP. None of the physicians started metformin for patients whose test results were in the prediabetic range, although one mentioned that a patient with prediabetes was already using metformin and a few acknowledged that metformin might be an option for such patients. One physician said, “I know there is some data talking about using metformin early on people like this. I don’t like to add more medications than I have [to]. So I don’t typically do that until the A1C starts to get up to closer to 6.5.”

## Discussion

Through CSR interviews among primary care physicians of a large academic medical center we found that screening for T2DM was appropriately targeted to patients with major risk factors for T2DM, including a history of prediabetes, older age, and being overweight or obese. The main reasons for not screening for T2DM included physicians’ knowledge of previously normal results of a screening test for T2DM and clinical encounters for reasons other than an HME. While physicians usually interpreted screening results correctly and consistently communicated these results to patients, they never recommended evidence-based treatments to patients with prediabetes, such as Diabetes Prevention Program participation or use of metformin.

One contributor to high rates of undiagnosed prediabetes and T2DM may be screening among only patients with certain risk factors. Consistent with prior studies [31], physicians in our study targeted screening to individuals with certain major risk factors for T2DM such as obesity or a previously abnormal screening test result. However, our findings suggest that physicians may miss opportunities to screen older individuals who lack other risk factors for T2DM. Specifically, although “older age” was cited as reason for screening in over one-third of the discussed encounters, all of the patients discussed during the CSR interviews were old enough to meet ADA criteria for screening for T2DM. Further, in our sample the mean age was actually lower among patients who were screened for T2DM compared with patients who were not screened (58 years versus 61 years). One practical strategy to help clinicians consistently identify patients with major risk factors for T2DM would be to integrate age-based clinical reminders into the EHRs, an approach that has been shown to improve targeting of other types of clinical preventive services [32, 33].

A clinical encounter for a reason other than an HME was a common reason why physicians did not screen for T2DM, which suggests additional missed opportunities to screen at-risk patients for T2DM. While some physicians mentioned intentionally deferring screening to future HMEs, others felt they simply did not have the time to address preventive health issues in the context of problem-focused encounters. Primary care clinicians have been increasingly encouraged to perform opportunistic screenings during problem-focused and urgent care encounters [23, 28], and practice innovations could offer opportunities for other members of primary care teams to share responsibility for screening patients for T2DM when they seek primary care for a broader range of reasons [34].

While the primary care physicians we interviewed nearly always correctly interpreted results of screening tests for T2DM, their responses to these screening tests suggest many patients may not be receiving evidence-based preventive recommendations and treatments. Specifically, physicians never referred patients with prediabetes to a DPP or initiated treatment with metformin. This is consistent with prior studies, which show that physicians often fail to respond to abnormal screening test results [27, 35]. For example, one previous study found less than 1% of patients with prediabetes were prescribed metformin, and only 5% were referred to or attended health education, wellness, or lifestyle programs [35, 36]. Educational initiatives to increase physician awareness of the benefits of the DPP and metformin could be considered. However, because primary care physicians often report being over-burdened with preventive health tasks [37] an alternative strategy might be to reflexively target patients with prediabetes for education about strategies to prevent T2DM.

### Limitations

First, we conducted our study in a single academic center and our findings may not generalize to all clinical environments. Second, it is possible that physicians with a particular interest in screening for T2DM may have been more likely to participate in the study, and there were no nurse practitioners or physician assistants who chose to participate. Third, none of the patients discussed in the CSR interviews were found to have T2DM through screening, thus limiting our insight into how physicians interpret and respond to such results. Fourth, physicians’ responses could have been influenced by recall or social desirability biases, though physician use of the EHR during interviews and our inquiries about recent visits by preclinical medical students aimed to mitigate the potential for such biases. Further, although physician access to the EHR at the time of the interview was intended to prompt recall of the encounter, it is possible that some physicians may have used EHR data to justify their decisions to screen or not screen a patient for T2DM. Finally, the USPSTF issued an updated recommendation for screening for T2DM following our study period, which could lead to changes in physician screening practices that we could not capture during this study.

## Conclusions

Our study provides new insight into T2DM screening practices among primary care physicians. We identified factors that may contribute to high rates of undiagnosed T2DM and prediabetes and thus could limit the impact of recent national initiatives aimed at reducing the public health burden of T2DM and its complications. These factors could be addressed through systems-based strategies to increase the early identification and evidence-based treatment of patients with abnormal screening tests for T2DM. More research is needed to determine whether such strategies can increase the early identification of at-risk patients and better connect these patients with evidence-based treatments.

## Additional file

Additional file 1: Chart-stimulated recall interview guide. (DOCX 20 kb)

## Abbreviations

ADA: American Diabetes Association
AMA: American Medical Association
CDC: Centers for Disease Control and Prevention
CSR: Chart-Stimulated Recall
EHR: Electronic Health Record
FBG: Fasting Blood Glucose
HbA1c: Hemoglobin A1c
HME: Health Maintenance Examination
OGTT: Oral Glucose Tolerance Test
PCP: Primary Care Provider
RV: Return Visit
T2DM: Type 2 Diabetes Mellitus
UMHS: University of Michigan Healthcare System
USPSTF: United States Preventive Services Task Force
