# Supplementary material for: Understanding type 2 diabetes mellitus screening practices among primary care physicians: a qualitative chart-stimulated recall study
Source: BMC Fam Pract. 2017 Apr 4;18:50. doi: 10.1186/s12875-017-0623-3 (PMC5381083; doi:10.1186/s12875-017-0623-3)
Supplement: Additional file 1: — Chart-stimulated recall interview guide. (DOCX 20 kb) [file 12875_2017_623_MOESM1_ESM.docx]

**Additional file 1: Chart-stimulated recall interview guide**

1. Introduce self

Hello, my name is [name of interviewer]. I am part of the team that is trying to understand type 2 diabetes mellitus (T2DM) screening practices in University of Michigan Health System primary care clinics.

2. Introduce the project and explain the purpose of the interview:

- We know this is a lot of variability in T2DM screening practices among primary care providers. The purpose of this research is to try to understand the decision making process around T2DM screening practices.
- We are interviewing primary care providers throughout UMHS.
- During this interview, you will have access to the Electronic Health Record. You will be asked to review specific clinical encounters with patients you have seen over the past two weeks, and you will use these specific encounters to answer some questions about T2DM screening practices. If you do not recall a patient encounter, we will skip it and move on to the next.
- I want to encourage you to answer these questions honestly. There are NO right or wrong answers. We want to understand your experiences and the challenges you face when screening patients for T2DM. This information may support the development of better system to help providers screen patients for T2DM.
- This interview will take approximately 30-40 minutes and you will receive a $50 gift card for your time.

3. Describe how we will assure confidentiality, obtain consent, and answer any questions.

I want to let you know what will happen to the information you provide. I am recording this conversation so that we do not miss anything that you have to say. People working on this study will be the only ones who will use the interview recordings. We will take steps to ensure the information you provide remains confidential, which are detailed in the consent form. Individuals will not be named in any notes, report or summary. Before we begin, can you please take a few minutes to review and sign the consent form? It covers, in written form, what I have just explained to you. Your signature shows that you understand what the study is about and that you agree to be interviewed and recorded.

You don’t have to answer a question if you don’t want to, and your participation is completely voluntary. You may stop the interview at any time.

Do you have any questions for me before you review the consent form (then give consent form)?

I am going to turn on the recorder. ****TURN ON RECORDER****  The remainder of the conversation is being recorded.

**Chart-stimulated recall**

I would like to start by asking you to open the chart for patient number [1-10].

*The interviewer provides the interviewee with a printed key linking patient medical record number (MRN) with a corresponding numerical value.*

Please take a moment to review this chart and let me know when you are done.

1. Do you recall this patient?
   - If no: We will skip this patient and move on to the next. *The interviewer repeats the above process, moving on to the next patient in numerical order.*
   - If yes: Proceed to question 2.
2. ***Did you screen this patient for T2DM at his/her visit?***

| **If “no”** | **If “yes”** | | |
| --- | --- | --- | --- |
| 1. Can you tell me about why this patient was not screened? 2. Are there characteristics about this patient that influenced your decision about T2DM screening?    - PROMPT: did this patient's age or co-morbidities influence your decision about whether to screen for T2DM? 3. Are there certain visit types where you specifically consider screening for T2DM?    - PROMPT: do you screen patients for T2DM at urgent visits, return visits or healthcare maintenance examinations? 4. Are there ways that the system -- for example, the EHR or medical staff -- could have helped you screen this patient for T2DM? | 1. Can you tell me about why the patient was screened?    - PROMPT, if needed: What factors influenced your decision to screen this patient for T2DM? 2. Are there certain visit types where you specifically consider screening for T2DM?    - PROMPT: do you screen patients for T2DM at urgent visits, return visits or healthcare maintenance examinations? 3. What screening test(s) did you order? 4. Why was this/were these particular screening test(s) selected? 5. Is the result of the test you ordered available in MiChart? (if “no”, ask “why not?”)    - PROMPT, if necessary: How would you interpret the result(s)? Is the result normal or abnormal? 6. Did you communicate the result(s) to the patient? | | |
|  | **If “no”** | **If “yes”** |  |
|  | 1. Do you plan to communicate these results in the future? 2. If yes: How will the result be communicated? What will the patient be told?   If no: Why won't the result be communicated? | 1. How were the results communicated to the patient?  - PROMPT, if necessary: Did you send a letter? Call the patient? Arrange a follow-up visit?  1. Were any recommendations made to the patient?  - PROMPT, if necessary: Were plans made for lifestyle changes or medication? |  |

***At least 4 patient charts will be discussed during the interview. For each chart, the interviewer will follow the above interview protocol.***

**Post-interview questions:**

1. Do you think that the patients we discussed are representative of your typical patient population?

1. What barriers get in the way of screening patients for T2DM?
2. Are there ways that your office staff (e.g. medical assistant) or electronic health record could help you screen for T2DM?
3. What are your thoughts about using metformin to treat prediabetes?
4. Is there anything I didn’t ask about that you think is important that would help you in screening patients for T2DM?
5. Do you have any questions about the interview or study?

Thank you so much for taking the time to speak with me today. Here is the $50 gift card for your participation.
